# Total, Bioavailable, and Free Vitamin D Levels and Their Prognostic Value in Pulmonary Arterial Hypertension

**DOI:** 10.3390/jcm9020448

**Published:** 2020-02-06

**Authors:** Maria Callejo, Gema Mondejar-Parreño, Sergio Esquivel-Ruiz, Miguel A. Olivencia, Laura Moreno, Isabel Blanco, Pilar Escribano-Subias, Angel Cogolludo, Joan Albert Barbera, Francisco Perez-Vizcaino

**Affiliations:** 1 Department of Pharmacology and Toxicology. School of Medicine, Universidad Complutense de Madrid, 28040 Madrid, Spain; maria.callejo@ucm.es (M.C.); gemondej@ucm.es (G.M.-P.); sesquive@ucm.es (S.E.-R.); mioliven@ucm.es (M.A.O.); lmorenog@med.ucm.es (L.M.); acogolludo@med.ucm.es (A.C.); 2 Ciber Enfermedades Respiratorias (Ciberes), 28029 Madrid, Spain; isabelclinic@gmail.com (I.B.); jbarbera@clinic.ub.es (J.A.B.); 3 Instituto de Investigación Sanitaria Gregorio Marañón (IISGM), 28007 Madrid, Spain; 4 Department of Pulmonary Medicine, Hospital Clínic-Institut d’Investigacions Biomèdiques August Pi i Sunyer (IDIBAPS), Universitat de Barcelona, 08036 Barcelona, Spain; 5 Department of Cardiology. 12 de Octubre University Hospital, School of Medicine, Universidad Complutense de Madrid, 28041 Madrid, Spain; 6 Ciber Enfermedades Cardiovasculares (CiberCV), 28029 Madrid, Spain

**Keywords:** pulmonary arterial hypertension, vitamin D, survival, prognosis

## Abstract

*Introduction*: Epidemiological studies suggest a relationship between vitamin D deficiency and cardiovascular and respiratory diseases. However, whether total, bioavailable, and/or free vitamin D levels have a prognostic role in pulmonary arterial hypertension (PAH) is unknown. We aimed to determine total, bioavailable, and free 25-hydroxy-vitamin D (25(OH)vitD) plasma levels and their prognostic value in PAH patients. *Methods*: In total, 67 samples of plasma from Spanish patients with idiopathic, heritable, or drug-induced PAH were obtained from the Spanish PH Biobank and compared to a cohort of 100 healthy subjects. Clinical parameters were obtained from the Spanish Registry of PAH (REHAP). *Results*: Seventy percent of PAH patients had severe vitamin D deficiency (total 25(OH)vitD < 10 ng/mL) and secondary hyperparathyroidism. PAH patients with total 25(OH)vitD plasma above the median of this cohort (7.17 ng/mL) had better functional class and higher 6-min walking distance and TAPSE (tricuspid annular plane systolic excursion). The main outcome measure of survival was significantly increased in these patients (age-adjusted hazard ratio: 5.40 (95% confidence interval: 2.88 to 10.12)). Vitamin D-binding protein (DBP) and albumin plasma levels were downregulated in PAH. Bioavailable 25(OH)vitD was decreased in PAH patients compared to the control cohort. Lower levels of bioavailable 25(OH)vitD (<0.91 ng/mL) were associated with more advanced functional class, lower exercise capacity, and higher risk of mortality. Free 25(OH)vitD did not change in PAH; however, lower free 25(OH)vitD (<1.53 pg/mL) values were also associated with high risk of mortality. *Conclusions*: Vitamin D deficiency is highly prevalent in PAH, and low levels of total 25(OH)vitD were associated with poor prognosis.

## 1. Introduction

Pulmonary arterial hypertension (PAH) is currently defined by an abnormal increase in pulmonary vascular resistance not due to left-heart, respiratory, or thromboembolic disease [1]. It is a disorder with a significant burden in terms of severity and prevalence, and it entails a poor prognosis. The etiology of the disease is complex and multifactorial, and it preferentially affects women, exerting substantial impact on their quality of life, i.e., reduced functional ability, greater oxygen requirements, and an increased risk of mortality [2]. Despite the advances in the knowledge of PAH, there is a need to improve its prevention and treatment [2,3]. Several non-invasive predictors are used to evaluate the prognosis of pulmonary hypertension (PH), i.e., New York Heart Association (NYHA) functional class, reduced 6-min walk test (6MWT), diffusing capacity for carbon monoxide (DLCO), and B-type natriuretic peptide (BNP) and the N-terminal fragment of pro-BNP (NT-pro-BNP) levels [4].

Vitamin D (vitD) is a fat-soluble vitamin that is obtained from dermal synthesis following exposure to sunlight or from the diet. VitD in the liver is converted into 25-OH-cholecalciferol (25(OH)vitD), the most abundant circulating form of vitD. Generally, 25(OH)vitD plasma measure serves as indicator of the vitD status. 25(OH)vitD is further metabolized in the kidney into the most active metabolite, 1-α-25,dihydroxycholecalciferol, also known as calcitriol. It plays a crucial role in the regulation of calcium and phosphorous metabolism, and its deficiency leads to bone diseases [5]. VitD is also involved in cellular growth, metabolism, and innate and adaptive immune responses [5,6]. There is no consensus on the threshold levels for 25(OH)vitD deficiency, its assessment, and its treatment, and clinical practice is inconsistent. However, even using conservative thresholds in population surveys, an important proportion of the global population shows vitD deficiency due to insufficient solar light exposure and/or reduced dietary intake [7].

In recent years, observational studies showed an association between low vitD levels and increased cardiovascular risk, respiratory diseases, and all-cause mortality [5,8,9,10,11,12]. However, new randomized controlled trials with moderate or high doses of vitD rendered dissimilar results. For instance, vitD supplements failed to demonstrate systemic antihypertensive effects or to reduce cardiovascular events [13,14,15], but succeeded in reducing asthma [16] and chronic obstructive pulmonary disease (COPD) [17] exacerbations in patients with baseline 25(OH)vitD levels lower than 25 nmol/L (10 ng/mL)

About 85% of total 25(OH)vitD is bound to vitD-binding protein (DBP), about 15% is bound to albumin, and only less than 1% is free [18]. Albumin-bound 25(OH)vitD dissociates rapidly and is also biologically available in tissues, and DBP acts as a reservoir for vitD. Thus, bioavailable 25(OH)vitD is defined as free 25(OH)vitD plus albumin-bound 25(OH)vitD [19]. Current classifications of vitD status are based on total 25(OH)vitD concentrations. However, several studies reported that bioavailable 25(OH)vitD may be a better biomarker for vitD status. For example, free and bioavailable 25(OH)vitD levels may be useful predictors for the prognosis of patients with coronary artery disease [20]. By contrast, other studies refuted this view [21,22].

Some preliminary evidence suggests that vitD deficiency is common in PAH [23,24,25,26]. Herein, we aimed to examine the total, bioavailable, and free 25(OH)vitD levels and their relationship with relevant clinical variables in PAH. The main hypothesis of the study was that lower levels of total 25(OH)vitD predicted reduced survival.

## 2. Experimental Section

### 2.1. Study Design and Participants

Human samples were provided from Biobank repositories, and the study was approved by the research ethics committees of Hospital Gregorio Marañón (REF. 123/16), the Spanish PH Biobank, and the Biobanco Vasco. Informed consent from donors was obtained in all cases. We carried out a multicenter, observational case–control study. The PAH cohort included all PAH patients with idiopathic, hereditary, and drug-induced PAH, whose plasma samples were deposited at the Spanish Biobank of PH at the IDIBAPS (Barcelona, Spain). It included 68 patients, but one was excluded because 25(OH)vitD could not be determined. According to international guidelines [4], at the time of sampling, PAH is defined in the Biobank as a mean pulmonary arterial pressure (mPAP) of more than 25 mm Hg, with pulmonary capillary wedge pressure (PCWP) less than 15 mm Hg. Samples from another control cohort were obtained from the Biobanco Vasco (Bilbao, Spain) which included plasma from 100 subjects, paired by sex with the PAH cohort; with no known cardiovascular disease (two were excluded because 25(OH)vitD could not be determined). No clinical data were available from the control group.

### 2.2. Measurements of Total 25(OH)vitD and Intact Parathyroid Hormone (iPTH)

Total 25(OH)vitD was measured using a chemiluminescence immunoassay (ADVIA Centaur^®^ VitD Total assay, Siemens Healthcare Diagnostics) which conforms with the National Institutes of Health CDC VitD Standardization Certification Program at the Clinical Biochemistry Service, Gregorio Marañon Hospital. Values below the detection limit of the technique (4.2 ng/mL) were found in 11 samples (all from the PAH cohort) and were replaced by the limit value divided by √2 (i.e., 2.97 ng/mL) as reported (https://analytics.ncsu.edu/sesug/2003/SD08-Croghan.pdf). VitD deficiency was defined as values of 25(OH)vitD below 20 ng/mL (equivalent to 50 nM), and severe deficiency was considered as values below 10 ng/mL. Plasma intact parathyroid hormone (iPTH) was measured by immunoassay using the same platform as above. This iPTH assay has standardization traceable to the World Health Organization’s standard preparation code 79/500. iPTH could not be measured in three control and 10 PAH samples. Values within 10–55 pg/mL were considered in the normal range.

### 2.3. Measurements of DBP and Albumin

Plasma DBP levels were measured using a commercial enzyme-linked immunosorbent assay (ELISA) kit, according to the manufacturer’s instructions (R&D Systems; Minneapolis, MN, USA). Values of DBP are expressed in µg/mL (250 µg/mL equal to 4 µM). Two PAH samples were discarded because DBP could not be determined. Albumin plasma concentrations were tested by the colorimetric Bromocresol Green (BCG) method (Sigma-Aldrich, MAK124). The intensity of the color, measured at 620 nm, is directly proportional to the plasma albumin concentration. Albumin levels are expressed in g/dL (0.1 g/dL is equivalent to 15 µM).

### 2.4. Calculation of Bioavailable 25(OH)vitD and Free 25(OH)vitD

Plasma bioavailable and free 25(OH)vitD were calculated using total 25(OH)vitD, DBP concentration, albumin levels, and affinity constants for albumin and DBP, as previously reported [27]. Total 25(OH)vitD can be defined as follows: total 25(OH)vitD = free 25(OH)vitD + albumin-bound 25(OH)vitD + DBP-bound 25(OH)vitD. Free 25(OH)vitD can be assessed via an indirect method, using a formula, which was first described by Bikle et al. [28] and later used by many authors [20,29].

$$\mathrm{Free}\;25\left(\mathrm{OH}\right)\mathrm{vitD}\;=\;\frac{\mathrm{Total}\;25\left(\mathrm{OH}\right)\mathrm{vitD}}{1\;+\;\mathrm{Kalb}\text{×}\mathrm{albumin}\;+\;\mathrm{KDBP}\times \mathrm{DBP}}$$

The vitD bioavailable concentration can also be calculated using the following mathematical formula:

Bioavailable 25(OH)vitD=[(Kalb × albumin)+1] × Free 25(OH)vitD

Kalb is the affinity constant for 25(OH)vitD and albumin binding (6 × 10^5^ M^−1^), whereas KDBP is the affinity constant for 25(OH)vitD and DBP binding (7 × 10^8^ M^−1^). Total and free 25(OH)vitD, albumin concentration, and DBP levels are expressed in mol/L.

### 2.5. Clinical Variables

We obtained the following clinical data of the PAH patients described above from the Spanish Registry of PAH (REHAP) [30] which is linked to the Biobank: PAH subgroup, date of birth, sex, weight, height, date of death, date of lung transplantation, and whether the patient was alive on 31 January 2017. The following parameters were obtained from the REHAP at the nearest date within six months of the date of plasma sampling for the Biobank: New York Heart Association functional class, diffusing capacity of the lung for carbon monoxide (DLCO), 6-min walking distance (6MWD), BNP, pro-BNP, cardiac catheterization (mPAP, PCWP, right-atrial pressure, cardiac output, cardiac index), parameters from echo (TAPSE, systolic PAP, myocardial performance or TEI index), oxygen, and drug therapy. In order to analyze if vitD levels affect the prognosis of PAH patients, we categorized all patients into two groups above or below the median from total (7.17 ng/mL), bioavailable (0.91 ng/mL), free (1.53 pg/mL) 25(OH)vitD, and DBP (337.3 µg/mL). The predefined main endpoint was an increased survival or a reduction in mPAP for total 25(OH)vitD levels. However, there were very few data from right-heart catheterizations (<35%) in the registry within the predefined time frame chosen of six months. Thus, survival was finally the single main endpoint of the study. The non-invasive risk score [4], a simplified version of the 2015 European Society of Cardiology (ESC) and the European Respiratory Society (ERS) risk assessment score, was calculated using three noninvasive low-risk criteria: NYHA functional class I–II, 6MWD < 440 m, BNP < 50 ng/L, or NT-proBNP < 300 ng/L [31].

### 2.6. Statistical Analyses

Analyses were performed using GraphPad Software v7 (GraphPad Software Inc., San Diego, CA, USA). All data were analyzed with non-parametric statistics. Two-sample comparisons were analyzed using Mann–Whitney test, and data are presented as scatter plots and medians. Multiple-sample comparisons, NYHA functional class, risk assessment, and percentage of patients were analyzed by chi-square for trends, and data are represented as tables of contingency. Survival curves were analyzed using the Kaplan–Meier method and compared by the log-rank test. Treatments in PAH patients were compared using Fisher’s exact test. The Cox proportional hazard model was used to assess hazard ratios of the survival, i.e., the main outcome, and compared with the Wald test using Stata (version 15). Unadjusted and age-adjusted hazard ratios were calculated. A *p-*value less than 0.05 was considered statistically significant.

## 3. Results

### 3.1. Total 25(OH)vitD Plasma Levels

**Figure 1:**
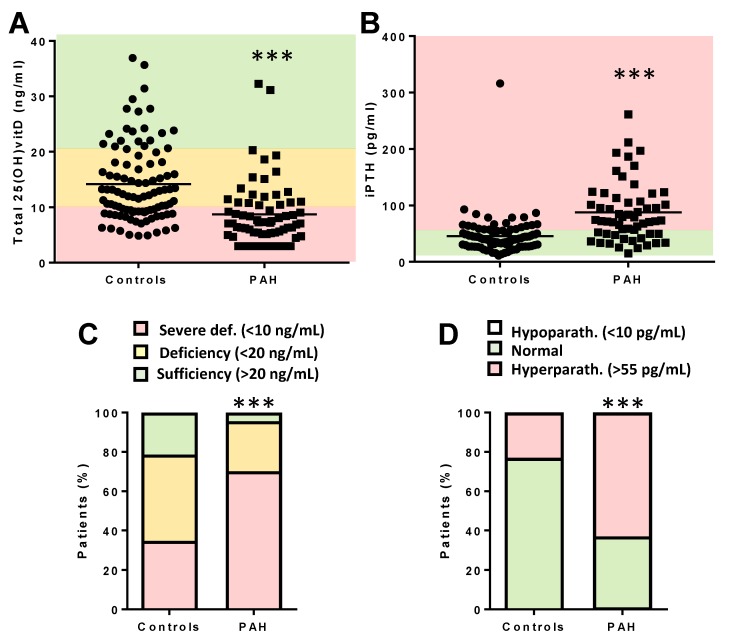
PAH patients present decreased total 25-hydroxy-vitamin D (25(OH)vitD) and increased intact parathyroid hormone (iPTH). (**A**) Total 25(OH)vitD and (**B**) iPTH plasma levels from controls and PAH patients. Colors indicate the ranges of levels as follows: for panels A and C, red encodes severe deficiency (<10 ng/mL), yellow encodes moderate deficiency (10–20 ng/mL), and green encodes sufficiency (>20 ng/mL); for panels B and D, green encodes normal levels (10–55 pg/mL) and red encodes hyperparathyroidism (>55 pg/mL). (**C**) Percentage of patients according to total 25(OH)vitD levels. (**D**) Percentage of patients according to iPTH range. Results in panels A and B are presented as scatter plots and medians; *** indicates *p* < 0.001 vs. controls, Mann–Whitney test. In panels C and D, *** denotes *p* < 0.001 vs. controls, chi-square test for trend.

The characteristics of PAH patients at diagnosis are shown in Table 1. Plasma sampling for the Biobank was carried out 3.8 (0.9) years (median and interquartile range (IQR)) after diagnosis. The control group, comprising 98 sex-matched patients without known cardiovascular disease, showed lower than recommended total 25(OH)vitD plasma levels (median (IQR): 12.27 (9.11–18.08) ng/mL; Figure 1A). Nevertheless, the group with PAH had significantly lower total 25(OH)vitD values (7.17 (5.15–10.84) ng/mL, Figure 1A) than controls. The levels were similar for the idiopathic and heritable PAH subgroups (6.97 (5.09–10.45), *n* = 58, and 7.30 (7.04–11.44), *n* = 7, respectively) and higher in the only two patients that had drug-induced PAH (11 and 15 ng/mL). The deficiency was present in both women (7.49 (5.46–10.84) ng/mL, *n* = 53) and men (5.93 (3.51–9.59) ng/mL, *n* = 14) with PAH (*p* > 0.05 vs. women, Mann–Whitney test). Therefore, 35% and 70% of the control subjects and PAH patients, respectively, had severe deficiency (Figure 1C). As expected, severe vitD deficiency in PAH was accompanied by significantly higher levels of iPTH than in the control group (Figure 1B). Thus, 70% of PAH patients showed hyperparathyroidism (i.e., iPTH > 55 pg/mL, Figure 1D).

### 3.2. Plasma DBP, Albumin, and Calculated Bioavailable and Free 25(OH)vitD

**Figure 2:**
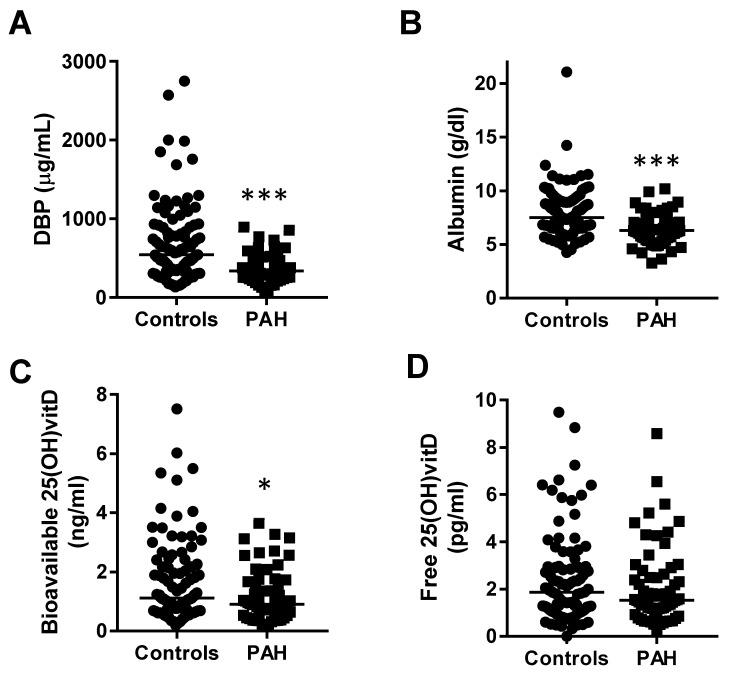
VitD-binding protein (DBP), albumin, and bioavailable but not free 25(OH)vitD are decreased in PAH patients. (**A**) Plasma DBP concentration, (**B**) albumin, (**C**) calculated bioavailable 25(OH)vitD, and (**D**) calculated free 25(OH)vitD from controls and PAH patients. Data are represented as scatter plots and medians. * and *** indicate *p* < 0.05 and *p* < 0.001 vs. controls, respectively, Mann–Whitney test.

PAH patients showed lower concentration of DBP (median (IQR): 337.3 (258.5–443.8) µg/mL; Figure 2A) than controls (median (IQR): 542.4 (330.9–893) µg/mL; Figure 2A). Plasma albumin concentration was also significantly lower in PAH patients (median (IQR): 6.31 (5.41–7.05) g/dL) than controls (median (IQR): 7.5 (6.51–9.06) g/dL; Figure 2B). Bioavailable and free 25(OH)vitD were calculated based on total 25(OH)vitD, albumin, and DBP levels. We also found that PAH patients presented lower bioavailable 25(OH)vitD (median (IQR): 0.91 (0.64–1.46) ng/mL; *n* = 65) than controls (median (IQR): 1.12 (0.71–2.19) ng/mL; *n* = 98, Figure 2C). Two PAH samples were discarded in this analysis because DBP could not be measured. By contrast, free 25(OH)vitD levels were similar in both groups (Figure 2D).

**Figure 3:**
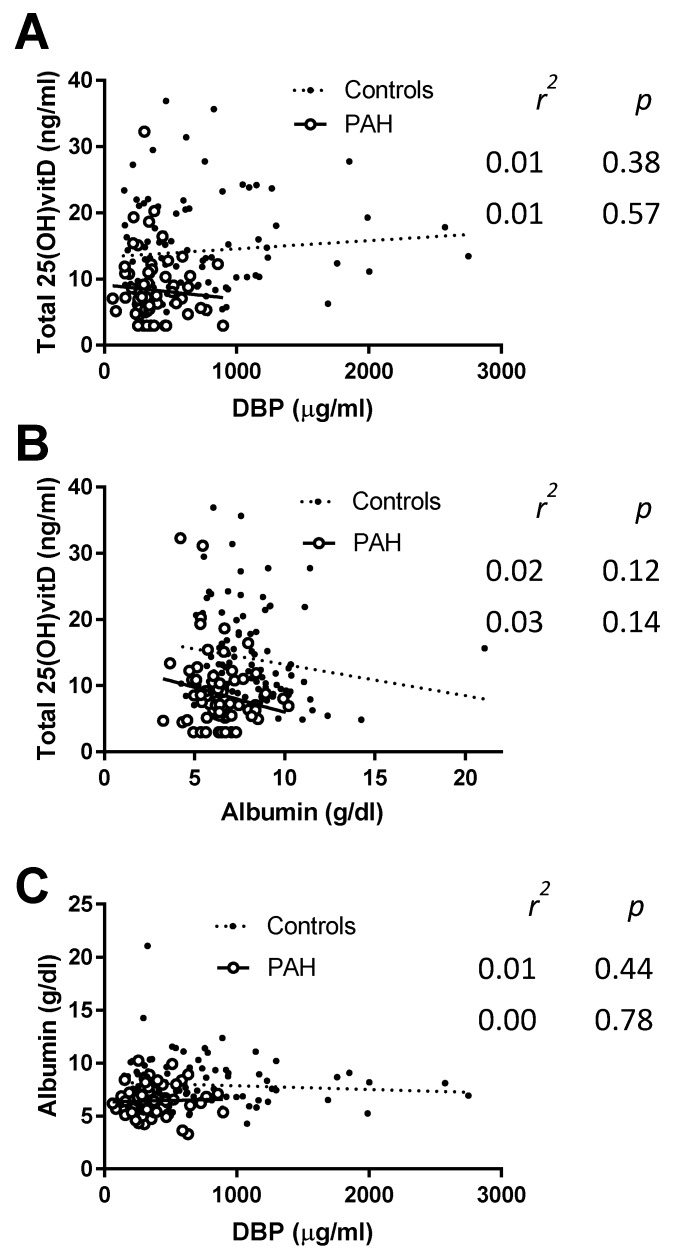
VitD-binding protein (DBP), albumin and total 25(OH)vitD plasma concentrations are independent variables. Correlations between (**A**) DBP and total 25(OH)vitD, (**B**) albumin and total 25(OH)vitD, and (**C**) DBP and albumin in controls and in PAH patients. The calculated Pearson *r^2^* and *p* are shown in each panel.

We analyzed possible relationships between total 25(OH)vitD, DBP, and albumin. However, we found no correlation among these parameters in either controls or PAH patients, indicating that these three variables were independent (Figure 3).

### 3.3. Total 25(OH)vitD Levels, Clinical Parameters, and Survival

**Figure 4:**
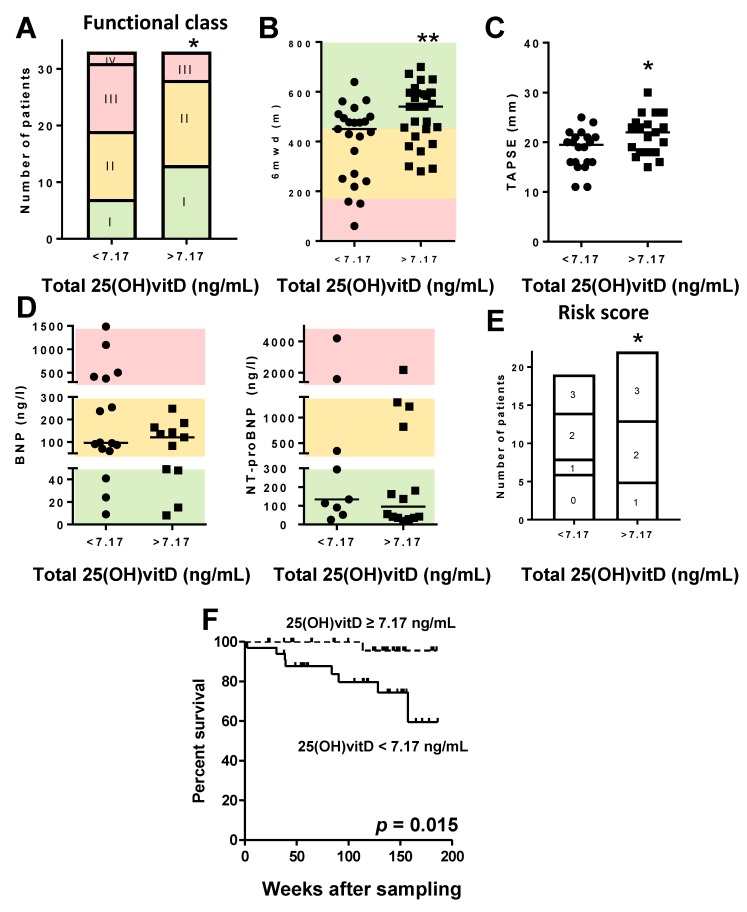
PAH patients with lower total 25(OH)vitD levels present worse prognosis. PAH patients were categorized according to total 25(OH)vitD levels (above vs. below the median (7.17 ng/mL) in the cohort). (**A**) NYHA functional class; (**B**) 6-min walking distance test (6MWD); (**C**) TAPSE; (**D**) BNP (left) and NT-proBNP (right). Colors in panels A, B, and D identify the ranges of these biomarkers considered of low (green), intermediate (yellow), or high (red) risk according to ERS/ESC guidelines for PAH. (**E**) Non-invasive risk score showing the number of patients with zero, one, two, or three low risk factors. In panels A and E, * denotes *p* < 0.05, chi-square test for trend. In panels B and D, data are scatter plots and medians; * *p* < 0.05 and ** *p* < 0.01 Mann–Whitney test. (**F**) Kaplan–Meier analysis of survival in PAH patients with total 25(OH)vitD levels above vs. below the median (7.17 ng/mL); *p* < 0.05, log-rank test.

We compared clinical variables (Table S1, Supplementary Materials) and pharmacological treatments (Table S2, Supplementary Materials) in PAH patients with total 25(OH)vitD plasma values below versus patients above the median (7.17 ng/mL) of this cohort. Patients below the median had more advanced functional class (Figure 4A). The exercise capacity, assessed as the 6MWD, and the right-ventricular systolic function, measured at echocardiography (TAPSE), were significantly lower in patients with total 25(OH)vitD levels below the median (Figure 4B,C). BNP levels were available for some patients, while NT-proBNP levels were available for other patients depending on the hospital. Despite these parameters not being significantly different (*p* > 0.05, Mann–Whitney test; Figure 4D), the proportion of patients at high risk according to BNP/NT-proBNP cut off levels as defined by ESC/ERS Guidelines (i.e., BNP > 300 ng/mL or NT-proBNP > 1400 ng/mL) was significantly higher (*p* < 0.05, Fisher chi-square for trend) in patients with total 25(OH)vitD levels below the median. Patients with higher 25(OH)vitD had significantly lower age and for all other hemodynamical parameters; follow-up data were scarce in the registry within six months of sampling and there were no significant differences (Table S1, Supplementary Materials).

We also analyzed the non-invasive risk score [31] based on the number of low-risk criteria in all PAH patients with total 25(OH)vitD above versus below the median. PAH patients with total 25(OH)vitD above the median (7.17 ng/mL) presented lower risk of death (Figure 4E). Remarkably, survival after plasma sampling, which was the main outcome measure of the study, was significantly increased (*p* < 0.01) in these patients (hazard ratio: 7.96 (95% confidence interval: 2.99 to 21.16)). Because there was a strong age unbalance between the patients with total 25(OH)vitD levels above versus those below the median, we carried out a Cox proportional hazard model adjusted for age. The age-adjusted hazard ratio was 5.40 (95% confidence interval: 2.88 to 10.12). The Kaplan–Meier analysis (Figure 4F) showed a significant reduced survival (*p* = 0.015) in patients with total 25(OH)vitD levels below the median. Treatments were similar in the two groups except for the use of long-term oxygen therapy, which was more frequent in PAH patients with lower total 25(OH)vitD (Table S2, Supplementary Materials).

### 3.4. DBP, Bioavailable and Free 25(OH)vitD, and Clinical Outcomes

**Figure 5:**
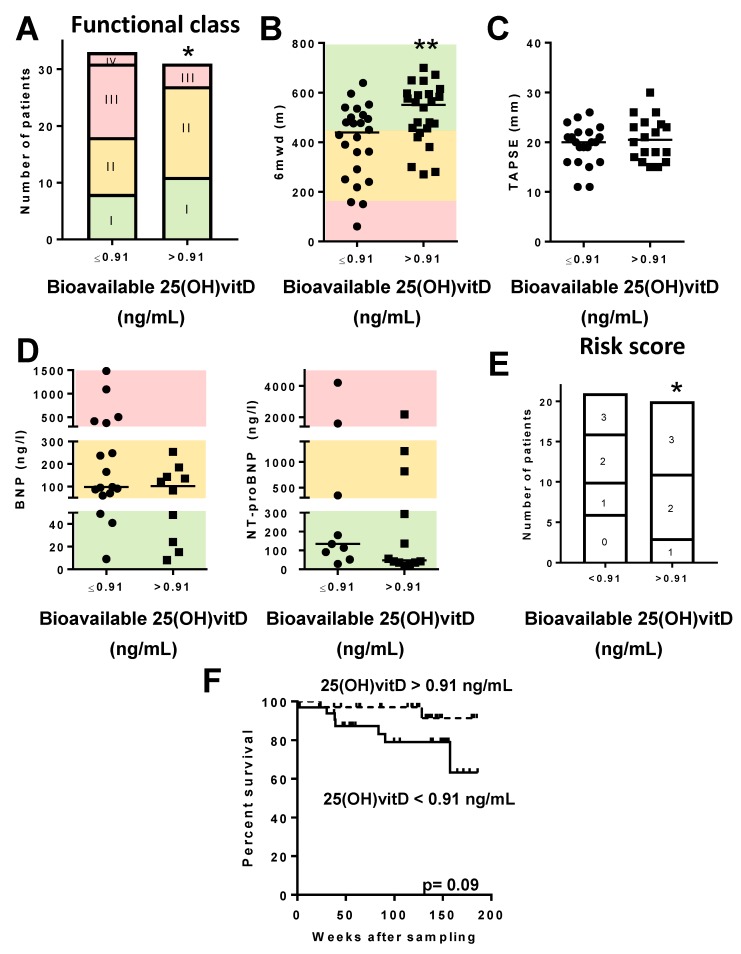
Bioavailable 25(OH)vitD levels in PAH patients and prognosis. PAH patients were categorized according to bioavailable 25(OH)vitD levels (above vs. below the median (0.91 ng/mL) in the cohort). (**A**) NYHA functional class; (**B**) 6-min walking distance test (6MWD); (**C**) TAPSE; (**D**) BNP (left) and NT-proBNP (right). Color codes are as in Figure 4. (**E**) Non-invasive risk score showing the number of patients with zero, one, two, or three low risk factors. In panels A and E, * denotes *p* < 0.05, chi-square test for trend. In panels B and D, data are scatter plots and medians, ** *p* < 0.01 Mann–Whitney test. (**F**) Kaplan–Meier analysis of survival in PAH patients with bioavailable 25(OH)vitD levels above vs. below the median (0.91 ng/mL); *p* = 0.09, log-rank test.

We also compared the clinical variables (Table S1, Supplementary Materials), the survival, and the pharmacological treatments (Table S2, Supplementary Materials) as a function of the DBP, albumin, and bioavailable and free 25(OH)vitD levels in plasma. For this purpose, we categorized all PAH patients into two halves: those with DBP levels above and those below the median (337.3 µg/mL). A similar categorization was made for albumin (median 6.31 g/dL), as well as for bioavailable (median 0.91 ng/mL) or free 25(OH)Vit (median 1.53 pg/mL). The clinical variables, the survival, and the therapies were similar in the two groups with low vs. high DBP (Tables S1 and S2, Supplementary Materials) However, patients with calculated bioavailable 25(OH)vitD below the median had significantly more advanced functional class (Figure 5A) and significantly lower 6MWD (Figure 5B). Moreover, the proportion of patients at high risk according to BNP/NT-proBNP cut off levels was significantly higher (*p* < 0.05, chi-square for trend) in PAH patients with bioavailable 25(OH)vitD levels below the median (Figure 5D). However, no differences were observed in TAPSE values according to bioavailable 25(OH)vitD levels (Figure 5C). Remarkably, PAH patients with bioavailable 25(OH)vitD levels over 0.91 ng/mL showed lower non-invasive risk score of mortality (Figure 5E), but the analysis of mortality did not show differences (*p* = 0.09, Figure 5F). For all the other clinical variables analyzed in this study, there were no significant differences (Table S1, Supplementary Materials). In addition, similar to total 25(OH)vitD results, long-term oxygen therapy was more frequent in PAH patients with lower bioavailable 25(OH)vitD (Table S2, Supplementary Materials).

**Figure 6:**
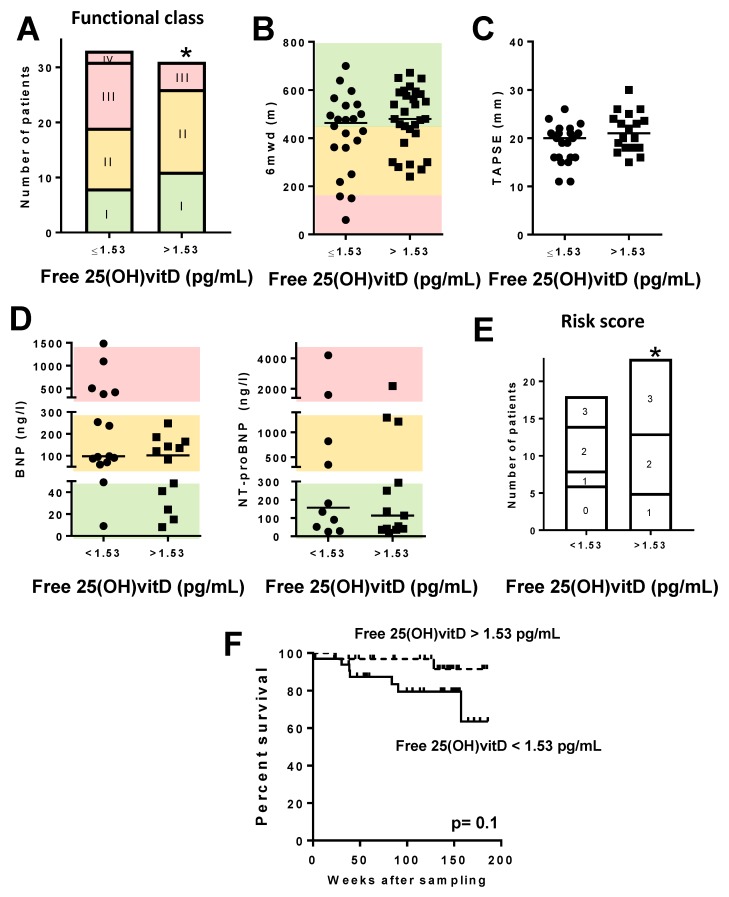
Free 25(OH)vitD levels in PAH patients and prognosis. PAH patients were categorized according to free 25(OH)vitD levels (above vs. below the median (1.53 pg/mL) in the cohort). (**A**) NYHA functional class; (**B**) 6 min walking distance test (6MWD); (**C**) TAPSE; (**D**) BNP (left) and NT-proBNP (right). Color codes are as in Figure 4. (**E**) Non-invasive risk score showing the number of patients with zero, one, two, or three low risk factors. In panels A and E, * denotes *p* < 0.05, chi-square test for trend. In panels B and D, data are scatter plots and medians; ** *p* < 0.01 Mann–Whitney test. (**F**) Kaplan–Meier analysis of survival in PAH patients with bioavailable 25(OH)vitD levels above vs. below the median (0.91 ng/mL); *p* = 0.10, log-rank test.

Regarding PAH patients with free 25(OH)vitD levels below vs. those above the median, we found that PAH patients with lower free 25(OH)vitD levels had a more advanced functional class (Figure 6A) and higher levels of BNP/NT-proBNP (*p* < 0.05, chi-square for trend, Figure 6D). There was also a significantly smaller proportion of males in the group with lower free 25(OH)vitD levels. However, there was no difference in any of the other clinical parameters analyzed or treatments (Tables S1 and S2, Supplementary Materials; Figure 6), except for a paradoxical borderline decrease in mPAP. However, the non-invasive risk score, which combines several risk factors, was significantly lower in patients with free 25(OH)vitD above the median (Figure 6E). Survival in the Kaplan–Meier analysis was also not significantly different (Figure 6F).

## 4. Discussion

The present study shows that up to 95% of patients with PAH have vitD deficiency (25(OH)vitD < 20 ng/mL) with 70% presenting severe deficiency (<10 ng/mL), and hyperparathyroidism (iPTH > 55 pg/mL). Moreover, we found that plasma DBP and bioavailable 25(OH)vitD are decreased in PAH. To our knowledge, this is the largest study analyzing the vitD status in PAH and the first one which analyzed the association of several plasma vitD biomarkers as total, bioavailable, and free 25(OH)vitD levels and DBP with the prognosis of PAH patients. Our results indicate that total 25(OH)vitD levels may have a prognostic value in PAH. When comparing bioavailable 25(OH)vitD in PAH patients vs. controls subjects, the magnitude of the difference and its statistical significance are smaller than for total 25(OH)vitD, and the significance is lost for free 25(OH)vitD. Likewise, the association of bioavailable 25(OH)vitD with prognosis is weaker than for total 25(OH)vitD, and it was even weaker for free 25(OH)vitD.

In line with the global pandemic of vitD deficiency [32,33], our control subjects present inappropriate vitD levels (median: 12.27 ng/mL) with 35% of them showing severe deficiency. Preliminary epidemiological studies suggested that vitD deficiency is more prevalent in PAH than in the general population. In fact, in our PAH cohort (*n* = 67), severe vitD deficiency was very common (median 7.17 ng/mL), in concordance with previous reports in small series (*n* = 22, 19, and 12) [23,24,26]. Values were very similar for idiopathic (class 1.1) and heritable PAH (class 1.2), and the sample of the drug-induced PAH (class 1.3) was too small to draw any conclusion. In a recent published study, 25(OH)vitD levels from PH patients with different etiologies were also analyzed as a single group [34].

Physiologically, decreased 25(OH)vitD levels result in proportionally increased PTH levels in order to maintain adequate serum calcium concentration. Several studies reported that secondary hyperparathyroidism and osteopenia are highly prevalent in PAH patients [24,35]. Therefore, inappropriate vitD status in PAH patients is likely to contribute to the observed elevated iPTH.

Tanaka et al. (23) also reported that total 25(OH)vitD levels negatively correlated with mPAP assessed by right-heart catheterization, with a significant positive correlation with cardiac output. According to the ESC/ERS guidelines for the diagnosis and treatment of PH and the World Health Organization [4], there are several factors that provide prognostic information and may be used to guide therapeutic decisions. Some of them, such as functional class, 6MWD, and BNP or NT-proBNP, are recommended to be assessed at each visit and are useful follow-up criteria. Functional class, despite its interobserver variability, is considered as one of the most powerful predictors of disease progression. In addition, exercise capacity measured by 6MWD test is also a strong predictor of mortality. BNP and NT-proBNP are not specific biomarkers for PAH, but they remain the only plasma biomarkers that are widely used in routine practice and in clinical trials. The non-invasive risk score is a simplified risk assessment tool that combines these three parameters, accurately discriminates prognostic groups, and predicts survival in PAH [31]. Notably, our results show that patients with total 25(OH)vitD levels above the median have better functional class, and they present higher exercise tolerance (around 90 m more) and lower BNP or pro-BNP levels. Likewise, the non-invasive risk score was also significantly different. TAPSE is another non-invasive prognostic factor often used to analyze ventricular function echocardiographically, which was also increased in patients with higher 25(OH)vitD. Other important determinants of prognosis such as right-atrial pressure, right-atrial area, cardiac index, ventilatory equivalents for carbon dioxide, and peak oxygen consumption were only available for few patients at the time of blood sampling and were not significantly different between the groups. Patients with more severe 25(OH)vitD deficiency had also higher requirements for long-term oxygen therapy. Altogether, these data suggest that patients with low 25(OH)vitD present worse prognosis. In fact, we found that these patients followed up after the blood sampling indeed had reduced survival.

VitD status in clinical practice is assessed by determining the total 25(OH)vitD concentration in serum or plasma. It is not the most active metabolite, but it is the major circulating form of vitD [5]. The majority of circulating 25(OH)vitD is tightly bound to DBP [18,19]. Thus, the assessment of total 25(OH)vitD levels principally measures the DBP-bound form, which is not biologically active [19,36,37]. Bioavailable 25(OH)vitD, which includes the free plus the easily released albumin-bound form, may be more representative to determine the vitD status. Diseases or conditions that affect the synthesis of DBP or albumin, thus, have a huge impact on the amount of circulating total 25(OH)vitD [27]. DBP and albumin are synthesized in the liver; hence, all patients with an impairment of liver function have alterations in their total vitD blood concentrations, while free vitD levels may remain mostly constant. Whether bioavailable or free 25(OH)vitD is a more accurate biomarker of vitD status than total 25(OH)vitD is disputed [18,19,20,21,22]. Remarkably, we found that PAH patients present lower plasma levels of both DBP and albumin than control patients. Since DBP and albumin act as a storage for total vitD, our results indicate that PAH patients show a lower reservoir capacity of vitD. Moreover, we also found that total 25(OH)vitD is not related to either DBP or albumin concentration; they are independent variables. This is consistent with the concept that vitD itself or its metabolites do not regulate DBP production [19,38]. Previous studies also reported lower serum albumin concentrations in PAH patients [39,40] and in PAH animal models [41], suggesting that reduced albumin may be a risk factor for PAH. Following the formulas given above, calculated free 25(OH)vitD is inversely related to DBP and albumin concentrations. Therefore, a decrease in plasma DBP and albumin as observed in PAH results in a relative increase in free 25(OH)vitD levels. Hence, in contrast to the data for total 25(OH)vitD levels, we did not find a significant difference in free 25(OH)vitD levels between controls and PAH patients. On the other hand, bioavailable 25(OH)vitD is inversely related to DBP and has a complex dependence on albumin. Thus, we still found a significant difference in bioavailable 25(OH)vitD levels between controls and PAH patients, but of smaller magnitude than for total 25(OH)vitD.

DBP knock-out mice, in which vitD metabolites are presumably all free and albumin-bound, do not show evidence of vitD deficiency and do not develop rickets. Thus, 25(OH)vitD bound to DBP does not contribute to the biological actions and DBP clearly serves as a critical circulating reservoir of vitD metabolites [19,42]. The mechanism leading to the low DBP concentration in PAH patients remains unknown. Patients with primary hyperparathyroidism have lower concentrations of both DBP and total 25(OH)vitD, without changes in free and bioavailable 25(OH)vitD. It might be possible that higher levels of iPTH inhibit hepatic DBP production [43,44]. Moreover, as noted earlier, albumin concentration does not correlate with total 25(OH)vitD levels.

Our results also show that bioavailable and free 25(OH)vitD were related with some clinical outcomes such as worse functional class and higher risk score. Additionally, bioavailable but not free 25(OH)vitD correlated with lower exercise capacity. By contrast, significant differences in survival were lost for both bioavailable and free 25(OH)vitD levels. Thus, total 25(OH)vitD may be associated more accurately with the progression of PAH, and it could be a more suitable biomarker for vitD status in PAH patients. Moreover, free or bioavailable 25(OH)vitD measurements are highly difficult, and, at this moment, there is only one immunoassay for the direct measurement of free 25(OH)vitD [45]. Some authors suggest that, if there is a correlation between bioavailable and/or free 25(OH)vitD with total 25(OH)vitD, it would only be necessary to measure total 25(OH)vitD [38].

In addition, the lack of activity of DBP-bound 25(OH)vitD was also questioned [36]. DBP can bind megalin, a receptor found in the plasma membrane of many epithelial cells. In the kidney, megalin acts as a cell surface receptor for DBP, and it internalizes DBP-bound 25(OH)vitD, a mechanism which is essential for 25(OH)vitD renal metabolism. Megalin is also highly expressed in the lung, and it is linked to the effects of transforming growth facto-β(TGF-β) [46]; however, it is not yet clear whether it plays a role on the pulmonary actions of vitD.

The reduced 25(OH)vitD plasma values that we found in patients with PAH does not establish a cause–effect relationship with the disease. Due to changes in lifestyle and environment, reduced outdoor activities, and inadequate sun exposure, vitD deficiency is a common phenomenon in the healthy population. Because PAH is a life-limiting disease, associated with weakness, fatigue, and exercise intolerance, PAH patients are more likely to have reduced sunlight exposure and, thus, become more susceptible to vitD deficiency. In fact, critically ill patients show a high prevalence of hypovitaminosis D [47]. Therefore, vitD deficiency may be a consequence of PAH. However, it is also reasonable that vitD-deficient states may worsen existing immune, metabolic, and cardiovascular dysfunctions [5,6], leading to the development of PAH in predisposed patients or to worse outcomes in patients with existing PAH. With the exception of idiopathic PAH, in all other forms of the disease, there is a factor known to be involved in its etiopathogeny, including mutations, systemic diseases, congenital heart defects, infections, drugs, and toxins. However, none of these factors by itself is able to trigger the disease, and the need for second hit(s) was proposed. vitD is involved in numerous processes of potential relevance in PAH, such as cell proliferation, differentiation, and apoptosis, cell adhesion, oxidative stress, angiogenesis, and immunomodulatory and anti-inflammatory activity [5]. Therefore, vitD deficiency might be one of these second hits. For instance, the prevalence of PAH in patients with systemic sclerosis is around 33%. In a cohort with systemic sclerosis, levels of 25(OH)vitD above 30 ng/mL had an incidence of echocardiographically elevated mPAP of 5% versus 40% in vitD-deficient patients [48]. In another cohort of patients with systemic sclerosis, subjects with vitD levels <30 ng/mL had higher systolic pulmonary arterial pressure than those with lower levels (34 vs. 25 mmHg) [49].

Restoration of vitD status would represent a very feasible health improving therapy for these patients. In fact, vitD supplements should be prescribed in any healthy or sick subject with deficient 25(OH)vitD to prevent osteomalacia [50]. Whether prognosis and symptoms of PAH patients improve after restoring vitD levels remains unclear. Remarkably, in a small uncontrolled cohort of PAH patients, restoring vitD levels by a cholecalciferol treatment at a dose of 50,000 IU weekly for three months improved the 6MWD by around 80 m and right-ventricular size but did not significantly reduce mPAP, measured by echocardiography (*p* = 0.07) and functional class [26]. Further randomized clinical trials are warranted. However, it would be prudent to choose only those patients with severe deficiency for future randomized studies in PAH patients in order to restore 25(OH)vitD values to normal range. Recently, some studies highlighted that an excess of vitD can cause calcified vasculopathy and valvulopathy, increase renin–angiotensin via hypercalciuria, and increase sympathetic activity [51,52]. In fact, vitD supplementation is used as a treatment in several pathologies regardless of baseline serum levels. This mistaken approach rendered dissimilar results; thus, vitD supplements failed to prevent cancer or to reduce cardiovascular events [53,54,55]. However, in patients with baseline vitD levels less than 25 nmol/L (10 ng/mL), supplements were beneficial in respiratory diseases, such as reducing asthma [16] and chronic obstructive pulmonary disease [17] exacerbations.

Our study presents some limitations. Right-heart catheterization was not available within the predefined six-month period around the moment of blood sampling for the biobank for most patients, and echocardiographic data were available for only 60% of them. Due to the low number of patients in hereditable (*n* = 7) and in drug-induced PAH (*n* = 2), all PAH patients were analyzed as a single group. Moreover, due to the retrospective nature of the trial, 25(OH)vitD levels were only measured once, and it remains unknown whether 25(OH)vitD levels changed over time or whether patients received vitD supplementation thereafter. The differences found cannot be explained by differences in the time of evolution of the disease from the diagnosis to the plasma sampling, i.e., a longer time after diagnosis would predict worse functional class and worse survival expectative. In fact, there was a trend for higher time of evolution in patients with total 25(OH)vitD above the median. However, we found a significantly lower age (median of 49 years) in the group with total 25(OH)vitD levels above the median compared to those with lower levels (58 years). The aging process itself predisposes to vitamin D deficiency due to a progressive decline in the cutaneous capacity to synthetize vitD and reduced exposure to sunlight [56]. This bias may have influenced the results because the five-year survival was reported to be higher in PAH patients aged 18–45 years (88%), while the survival rates were 63%, 56%, and 36% for patients in the groups 46–64, 65–74, and ≥75 years, respectively [57]. However, after adjusting for age, survival still remained significantly different for those with high vs. low levels of total 25(OH)vitD.

## 5. Conclusions

The present study demonstrates that total 25(OH)vitD, rather than bioavailable or free 25(OH)vitD, is a potential predictor of adverse outcomes in PAH patients. Given the high prevalence of vitD deficiency in PAH population, it seems reasonable that serum total vitD levels should be regularly assessed. Further studies are required to clarify whether our findings have potential clinical implications.

## Figures and Tables

**Table 1:** Patient characteristics at diagnosis.

	PAH	Controls
*N*	67	98
Idiopathic	58	-
Heritable	7	-
Drug-induced	2	-
Age (years)	47 (31.5–62)	39 (29–47)
Female (%)	53 (79%)	80 (81%)
mPAP (mm Hg)	52 (43–61)	-
Cardiac index (L/min/m^2^)	2.2 (1.8–2.5)	-
DLCO (mL/min/mmHg)	64 (47.5–73.5)	-
6MWD (m)	412 (336–474)	-
Functional class (I, II, III, IV)	1, 23, 40, 3	-

Data are median (interquartilic range, IQR). PAH: pulmonary arterial hypertension; mPAP: mean pulmonary arterial pressure; DLCO: CO diffusing lung capacity; 6MWD: six-minute walk distance.

## Supplementary Materials

The following are available online at https://www.mdpi.com/2077-0383/9/2/448/s1: Table S1: Exercise capacity, hemodynamic and echocardiographic variables measured within 6 months of the plasma sampling in PAH patients with total, bioavailable and free 25(OH)vitD levels and DBP levels below versus above the median in this cohort, Table S2: Treatments in PAH patients with 25(OH)vitD levels below versus above the median in this cohort.
