# Supplementary material for: Total, Bioavailable, and Free Vitamin D Levels and Their Prognostic Value in Pulmonary Arterial Hypertension
Source: J Clin Med. 2020 Feb 6;9(2):448. doi: 10.3390/jcm9020448 (PMC7073767; doi:10.3390/jcm9020448)
Supplement: Supplementary file 1 [file jcm-09-00448-s001.pdf]

Supplementary Tables S1 and S2

Supplementary Table S1. Exercise capacity, hemodynamic and echocardiographic variables measured within 6 months of the plasma sampling in PAH patients with total, bioavailable and free 25(OH)vitD levels and DBP levels below versus above the median in this cohort.

**Total 25(OH)VitD**					
	25OHVitD <7.17 ng/ml		25OHVitD ≥ 7.17 ng/ml		p
	n	Median (IQR)	n	Median (IQR)	
Days from diagnosis to sampling	33	853 [181-2739]	34	2300 [775-3762]	0.162
Age at plasma sampling	33	59 [43.5-70]	34	48 [34-59.75]	**0.01**
Sex (M/F)	33	10/23	34	5/29	0.15
6MWD (m)	23	450 [250-500]	27	540 [420-596]	**0.009**
BNP	16	97 [62-406]	11	121 [48-165]	0.312
NT-Pro-BNP	9	134 [71-970]	14	96 [36-917]	0.460
mPAP (RHC) (mm Hg)	15	46 [39-54]	11	60 [40-66]	0.073
PCWP (mm Hg)	15	8 [7-10]	11	8 [6-11]	0.812
RAP (mm Hg)	15	6 [4-9]	11	7 [4-11]	0.419
CI (l/min/m^2)	15	2.14 [1.74-2.29]	11	2.30 [1.81-3.31]	0.276
TAPSE (mm)	20	19.5 [16-21]	19	22 [18,24]	**0.039**
sPAP (echo) (mm Hg)	22	70 [63.5,84.7]	20	63 [48.5,87.7]	0.290
Functional class (I, II, III, IV)	33	7, 12, 12, 2	33	13,15,5,0	**0.011**[1]
**Bioavailable 25(OH)VitD**					
	25OHVitD≤0.91 ng/ml		25OHVitD>0.91 ng/ml		p
	n	Median [IQR]	n	Median [IQR]	
Days from diagnosis to sampling	33	1058 [178-2986]	32	2061 [596.5-4366]	0.19

Age at plasma sampling	33	59[41-69]	32	48[37-61]	0.09
Sex (M/F)	33	9/24	32	5/27	0.36
6MWD (m)	24	439.5 [260.3-507.5]	24	550.5 [442.5-596.3]	**0.01**
BNP	17	98 [65-396.5]	10	102 [21.75-153.5]	0.18
NT-Pro-BNP	9	180 [101.5-1057]	12	360 [164.5-751]	0.58
mPAP (RHC) (mm Hg)	14	44 [38-54.5]	11	57 [40-63]	0.16
PCWP (mm Hg)	14	8 [7-10]	11	8 [6-11]	0.99
RAP (mm Hg)	15	7 [5-9]	11	7 [4-10]	0.95
CI ($l/min/m^2$)	15	2.14 [1.74-2.29]	10	2.2 [1.77-3.32]	0.39
TAPSE (mm)	21	20 [16-22]	17	21 [17-23.8]	0.44
sPAP (echo) (mm Hg)	23	65 [55-82]	18	72.5 [56.5-87.75]	0.37
Functional class (I, II, III, IV)	33	8,10,13,2	31	11,16,4,0	**0.01**[1]
**Free 25(OH)VitD**					
	25OHVitD≤1.53 pg/ml		25OHVitD>1.53 pg/ml		p
	n	Median [IQR]	n	Median [IQR]	
Days from diagnosis to sampling	33	1058 [225.5-3171]	32	2059 [596.5-4032]	0.31
Age at plasma sampling	33	56[38-68]	32	49[38-62]	0.25
Sex (M/F)	33	11/22	32	3/29	**0.03**
6MWD (m)	22	463 [332.5-536.3]	29	480 [400.5-586.5]	0.17
BNP	15	98 [70-416]	12	102 [28.25-159.5]	0.11
NT-Pro-BNP	10	298.5 [123-1012]	11	360 [113-550]	0.9
mPAP (RHC) (mm Hg)	14	43 [38-53]	11	60 [40-63]	**0.04**
PCWP	14	8	11	9	0.56

(mm Hg)		[6.75-10]		[6-11]	
RAP<br>(mm Hg)	15	7<br>[4-9]	11	7<br>[4-10]	0.85
CI<br>(l/min/m^2)	14	2.145<br>[1.73-2.29]	11	2.1<br>[1.81-3.31]	0.46
TAPSE<br>(mm)	21	20<br>[16-21.5]	17	22<br>[18-24.5]	0.07
sPAP (echo)<br>(mm Hg)	23	67<br>[55-83]	18	66.5<br>[54.5-87]	0.99
Functional class<br>(I, II, III, IV)	33	8,11,12,2	31	11,15,5,0	**0.03**
**DBP**					
	DBP ≤ 337.3 µg/ml		DBP > 337.3 µg/ml		p
	n	Median [IQR]	n	Median [IQR]	
Days from diagnosis to sampling	34	1533<br>[301.8-3145]	32	1273<br>[316-3355]	0.88
Age at plasma sampling	34	53[42-66]	32	54[34-67]	0.51
Sex (M/F)	34	6/28	32	8/24	0.55
6MWD<br>(m)	24	475.5<br>[300-588.3]	25	480<br>[371.5-559]	0.91
BNP	10	103 [36.75-284.8]	17	98 [71.5-242.5]	0.64
NT-Pro-BNP	16	326.5 [134.5-542.5]	6	263.5 [74.75-1664]	0.84
mPAP (RHC)<br>(mm Hg)	13	53<br>[40-61.5]	12	43<br>[35.5-55.5]	0.15
PCWP<br>(mm Hg)	13	9<br>[7.5-11]	12	8<br>[6-9.75]	0.29
RAP<br>(mm Hg)	13	6<br>[4-13.5]	13	7<br>[5-8.75]	0.97
CI<br>(l/min/m^2)	12	2.22<br>[1.89-3.16]	13	1.96<br>[1.67-2.26]	0.11
TAPSE<br>(mm)	22	21.5<br>[17.5-23.7]	16	19.5<br>[16.21]	0.26
sPAP (echo)<br>(mm Hg)	20	66.5<br>[55.25-86.25]	21	67<br>[55-79.5]	0.85
Functional class<br>(I, II, III, IV)	33	9,16,7,1	32	10,11,10,1	0.76

6MWD: six-minute walk distance; BNP: brain natriuretic peptide; mPAP: mean pulmonary arterial pressure by right heart catheterization; PCWP: pulmonary wedge pressure; RAP: right atrial pressure; CI: cardiac index; TAPSE: tricuspid annular plane systolic excursion; sPAP: systolic pulmonary arterial pressure by echocardiography. RHC right heart catheterization. [1]Chi-square test for trend. Sex (M: male; F: female), Fischer's test.

Supplementary Table S2. Treatments in PAH patients with 25(OH)vitD levels below versus above the median in this cohort.

**Total 25(OH)vitD**			
	25OHvitD <7.17	25OHvitD ≥ 7.17	p
Oxygen	10 (30%)	2 (6%)	**0.01**
Anticoagulants	19 (58%)	24 (71%)	0.31
Diuretics	19 (58)	12 (35%)	0.08
Digoxin	6 (18%)	3 (9%)	0.30
CCBs	9 (27%)	10 (29%)	0.27
PDE5i	24 (73%)	24 (71%)	0.99
ERAs	16 (48%)	21 (62%)	0.33
Prostanoids	7 (21%)	9 (26%)	0.78
Monotherapy	14 (42%)	13 (38%)	0.81
Dual therapy	13 (39%)	11 (32%)	0.62
Triple therapy	4 (12%)	7 (20%)	0.51
Cuadruple therapy	1 (3%)	2 (6%)	0.99
Bioavailable 25(OH)vitD			
	25OHvitD ≤ 0.91 ng/ml	25OHvitD > 0.91 ng/ml	p
Oxygen	10 (30%)	2 (6%)	**0.02**
Anticoagulants	19 (57%)	22 (68%)	0.44
Diuretics	15 (45%)	14 (43%)	0.99
Digoxin	5 (15%)	4 (12%)	0.99
CCBs	9 (27%)	10 (31%)	0.78
PDE5i	22 (66%)	25 (78%)	0.40
ERAs	18 (54%)	17 (53%)	0.99
Prostanoids	5 (15%)	10 (31%)	0.15
Monotherapy	15 (46%)	11 (35%)	0.45
Dual therapy	13 (40%)	11 (35%)	0.80
Triple therapy	3 (9%)	7 (22%)	0.18
Cuadruple therapy	1 (3%)	2 (6%)	0.61

Free 25(OH)vitD			
	25OHvitD ≤ 1.53 pg/ml	25OHvitD > 1.53 pg/ml	p
Oxygen	9 (27%)	3 (9%)	0.11
Anticoagulants	19 (57%)	22 (68%)	0.79
Diuretics	17 (51%)	12 (37%)	0.32
Digoxin	5 (15%)	4 (12%)	0.99
CCBs	9 (27%)	10 (31%)	0.79
PDE5i	24 (72%)	23 (71%)	0.99
ERAs	19 (57%)	16 (50%)	0.62
Prostanoids	6 (18%)	9 (28%)	0.39
Monotherapy	14 (43%)	12 (37%)	0.80
Dual therapy	12 (37%)	12 (37%)	0.99
Triple therapy	5 (15%)	5 (16%)	0.72
Cuadruple therapy	1 (3%)	2 (6%)	0.61
DBP			
	DBP ≤ 337.3 µg/ml	DBP > 337.3 µg/ml	p
Oxygen	8 (23%)	5 (15%)	0.56
Anticoagulants	20 (58%)	22 (68%)	0.45
Diuretics	17 (50%)	12 (37%)	0.33
Digoxin	3 (8%)	6 (18%)	0.30
CCBs	8 (23%)	11 (34%)	0.42
PDE5i	27 (79%)	21 (65%)	0.27
ERAs	19 (55%)	16 (50%)	0.81
Prostanoids	8 (23%)	7 (21%)	0.99
Monotherapy	13 (38%)	14 (46%)	0.61
Dual therapy	15 (44%)	9 (30%)	0.31
Triple therapy	5 (14%)	5 (16%)	0.99
Cuadruple therapy	1 (2%)	2 (6%)	0.60
